# Association between social support and frequency of physical activity in adult workers

**DOI:** 10.47626/1679-4435-2022-786

**Published:** 2023-02-13

**Authors:** Thaísa Alves Penna, Vitor Barreto Paravidino, Fabiane Frota da Rocha Morgado, Bruno Ribeiro Ramalho de Oliveira, Geraldo de Albuquerque Maranhão-Neto, Sérgio Machado, Eduardo Lattari, Aldair J. Oliveira

**Affiliations:** 1 Programa de Pós-Graduação em Ciências da Atividade, Universidade Salgado de Oliveira, Niterói, RJ, Brazil; 2 Departamento de Epidemiologia, Instituto de Medicina Social, Universidade do Estado do Rio de Janeiro, Rio de Janeiro, RJ, Brazil; 3 Departamento de Educação Física e Desportos, Universidade Federal Rural do Rio de Janeiro, Seropédica, RJ, Brazil; 4 Kardiovize Study, International Clinical Research Centre, St. Anne’s University Hospital, Brno, Czech Republic

**Keywords:** physical exercise, social network, occupational categories, exercício físico, rede social, categorias de trabalhadores

## Abstract

**Introduction:**

The benefits of taking up physical activity are well established and social support has been identified as one of the main determinants of this behavior.

**Objectives:**

To investigate the association between social support and weekly frequency of physical activity in adults working at a public university in the state of Rio de Janeiro, Brazil.

**Methods:**

This is a cross-sectional population study with a convenience sample of 189 contract workers of both sexes, aged from 21 to 72 years (39.00 ± 11.43). The instruments employed were the short version of the International Physical Activity Questionnaire and the Social Support for Physical Activities Scale. Fisher’s exact test was used to estimate the distribution of physical activity frequency. Poisson regression was used for association analyses. The significance level was set at 5%.

**Results:**

A significant association was detected between social support and weekly physical activity frequency (p < 0.05). Social support for physical activity of moderate or vigorous intensity was associated with both weekly frequency of walking (odds ratio [OR]: 1.32; 95% confidence interval [95%CI]: 1.11-1.58) and weekly frequency of vigorous physical activity (OR: 1.34; 95%CI: 1.08-1.67). Additionally, people who reported receiving social support for walking were more likely to have increased weekly frequency of walking (OR: 1.22; 95%CI: 1.00-1.49).

**Conclusions:**

Social support for physical activity from relatives and friends is associated with weekly physical activity frequency. Notwithstanding, this association was stronger for weekly frequency of vigorous intensity physical activity.

## Abstract (pt)

**Introdução:**

Os benefícios advindos da adoção da atividade física estão bem consolidados, e o apoio social tem sido apontado como um dos principais determinantes desse comportamento.

**Objetivos:**

Investigar a associação entre apoio social e frequência semanal de atividade física em adultos trabalhadores de uma universidade pública do estado do Rio de Janeiro.

**Métodos:**

Tratou-se de um estudo transversal de base populacional, abarcando uma amostra de conveniência de 189 funcionários terceirizados de ambos os sexos, com idades entre 21 e 72 anos (39,00 ± 11,43). A versão curta do Questionário Internacional de Atividade Física e a Escala de Apoio Social para Atividades Físicas foram os instrumentos utilizados. Para estimar a distribuição da frequência em atividades físicas, foi aplicado o teste exato de Fisher. A regressão de Poisson foi utilizada para análise de associação. O nível de significância adotado foi de 5%.

**Resultados:**

Uma diferença significante foi encontrada entre apoio social e frequência semanal de atividade física (p < 0,05). O apoio social para atividade física de intensidade moderada ou vigorosa esteve associado tanto com frequência semanal de caminhada (razão de chances [OR, de *odds ratio*]: 1,32; intervalo de confiança de 95% [IC95%]: 1,11-1,58) quanto com frequência de atividade física vigorosa (OR: 1,34; IC95%: 1,08-1,67). Além disso, indivíduos que reportaram receber apoio social para caminhada tiveram mais chances de aumentar a frequência semanal de caminhada (OR: 1,22; IC95%: 1,00-1,49).

**Conclusões:**

O apoio social para atividade física advindo de familiares e amigos está associado à frequência semanal de atividade física. Contudo, a associação se mostrou mais evidente para frequência semanal de atividade física de intensidade vigorosa.

## INTRODUCTION

The benefits of a physically active life are well established. Spending time engaged in physical activities can reduce conditions that are harmful to health, such as cardiovascular diseases, hypertension, diabetes, obesity, and some types of cancer,^1-4^ resulting in better quality of life. However, this evidence has not been enough to change the population’s behavior, considering the high rates of physical inactivity observed.^5^

Several different factors influence people to perform or not perform physical activity, and it has been observed that social support is one of the most important.^6^ It can be understood as the resources provided by a person’s network of relationships that support performing an activity.^7^ Its influence derives from the fact that physical activity is a behavior that is generally linked to interactions that take place within a network of social connections, of which friends and family are a part.^8^

Social support has considerable impact on many aspects of life, with positive relationships with many different health outcomes, both physical and mental, especially so when evaluated in terms of people’s opinions of the social support they receive.^9^ The positive effects of social support and social networks on behavior and quality of life include helping people to cope with chronic diseases, the relations that patients develop with health services and health professionals, adherence to treatments, and adoption of new lifestyles.^10^

Social support has gained prominence in investigations of physical activity because it has proven relevant to promotion and stimulus of preventative health promotion practices.^8^ Some studies have demonstrated that people who receive social support for physical activity become more active than those who do not receive support.^11,12^ However, there are few investigations of this subject in less developed countries. The subject therefore needs to be investigated further, particularly in medium and low income countries, because the opportunity landscape is distinct from that found in more developed countries.

Another aspect to be taken into consideration is the type of population investigated. Previous studies suggest that people who work find it more difficult to adopt physically active behavior, since the working routine is the obstacle that members of this population most often report.^13^ As such, although the association between social support and physical activity is well established in the literature,^14^ additional new evidence is needed with regard to this section of the population. It should be noted that short weekly sessions of physical activity can have a positive impact on people’s health.^15^ Investigation of the frequency of physical activity is therefore important, because it can serve as an indicator of the extent to which workers can manage to be physically active, despite their time constraints.

This highlights the need to conduct studies that assess the relationship between social support and frequency of physical activity in adulthood, taking into account the types of social support (social support for walking and for physical activities of moderate or vigorous intensity) and the different types of physical activity (walking, moderate intensity, or vigorous intensity). Therefore, the objective of this study was to investigate the association between social support and weekly physical activity frequency in adult workers at a public university in the state of Rio de Janeiro, Brazil.

## METHODS

### STUDY DESIGN AND SAMPLE

This is a pilot for the Longitudinal Study of the Determinants of Physical Activity (ELDAF - Estudo Longitudinal dos Determinantes da Atividade Física), designed to investigate the psychosocial determinants of physical activity in technical and administrative workers at a public university in the Brazilian state of Rio de Janeiro. The convenience sample studied included technical and administrative contract workers of both sexes. Employees were excluded if they were on leave or were on secondment at another institution. The project was approved by the Research Ethics Committee (Ethics Appraisal Submission Certificate: 56224716.2.0000.5289) and all participants signed a free and informed consent form.

### DATA COLLECTION

Demographic, socioeconomic, physical activity, and social support data were collected by a team of eight investigators who had been trained in advance for this task. After their line managers had granted permission, each participant was invited individually by a member of our team and data were collected in each worker’s workplace, in a secluded area. After collection, data were double input, to enable correction of any input errors. The data collection process was conducted during two different periods: the first was from June to August of 2017 and the second was from June to July of 2018.

The technical and administrative workers were separated into two categories: manual workers and non-manual workers. The manual workers were those people whose jobs and tasks are performed manually and demand physical effort but little technical or scientific knowledge. In turn, the non-manual workers do not need to exert physical effort to carry out their jobs but do need technical or scientific knowledge to perform their tasks.

### PHYSICAL ACTIVITY

Physical activity was measured using the short form of the International Physical Activity Questionnaire (IPAQ).^16^ Weekly frequencies were estimated for walking and for moderate and vigorous intensity physical activity performed by participants.

### SOCIAL SUPPORT

The Social Support for Physical Activities Scale (SSPAS)^17^ was administered to enable investigation of social support. The SSPAS is based on a scale proposed by Sallis et al.^18^ and was developed for and validated with Brazilian adults. This instrument comprises two sections covering support from the family and from friends for physical activities (walking and moderate or vigorous intensity exercise), totaling 12 questions. Family members are defined as people who live with the respondent an0d therefore sleep and eat meals in the same residence; anybody else is defined as a friend. Participants reported the frequency with which family and friends exercised with them, invited them to exercise, or encouraged them to perform physical activity during the previous 3 months. The scale offers three response options: “never”, “sometimes”, and “always”. Scores for social support are calculated by summing the answers given for each section, varying from 0 to 6 points for walking or for moderate and vigorous intensity physical activity. The responses “sometimes” and “always” were analyzed together, so that people with a lower frequency of social support were still recorded as having received support. Participants were analyzed in two categories: individuals who did receive social support for physical activity and individuals who did not receive this resource.

### STATISTICAL ANALYSIS

Continuous variables are expressed as means and standard deviations and categorical variables are expressed as frequencies and percentages. Fisher’s exact test and Poisson regression were used to analyze associations between social support and weekly physical activity frequency. Odds ratios (OR) and their respective 95% confidence intervals (95%CI) were estimated for crude and adjusted models. Age, sex, and educational level were considered confounding variables and included in the adjusted model. Analyses were conducted using R, version 3.5.6.

## RESULTS

A total of 189 contract workers were recruited, 65.6% of whom performed manual activities as part of their jobs. Approximately 80% of the sample stated they received social support for walking, while 65.2% reported receiving social support for moderate or vigorous intensity physical activities (Table 1).

**Table 1:** Descriptive characteristics of the sample

Categorical variables	n (%)		
Sex			
Female	90 (49.0)		
Male	93 (51.0)		
Educational level			
Primary education	54 (28.6)		
Secondary education	93 (49.2)		
Higher education	42 (22.2)		
Job activities			
Manual work	124 (65.6)		
Non-manual work	65 (34.4)		
Weekly frequency	**PA, walking**	**PA, moderate**	**PA, vigorous**
0	59 (32.0)	33 (17.7)	37 (20.0)
1-3	58 (31.5)	72 (38.5)	31 (16.5)
4-5	43 (23.5)	41 (21.9)	64 (35.0)
6-7	24 (13.0)	41 (21.9)	53 (28.6)
Social support	**PA, walking**	**PA, moderate/vigorous**	
Has support	34 (21.8)	57 (34.8)	
Does not have support	122 (78.2)	107 (65.2)	
Continuous variable	**Mean (standard deviation)**		
Age	39.00 (11.43)		

PA = physical activity.

Table 2 lists the weekly frequency of physical activity according to the social support received. A significant association was only detected between social support for physical activity of moderate or vigorous intensity and frequency of walking activity (p = 0.004). Approximately 50% of workers who did not receive social support for physical activities of moderate or vigorous intensity did not report frequent walking physical activity, whereas 22.7% of those who did receive this support did not perform walking activity with frequency.

**Table 2:** Analysis of weekly frequency of physical activity, by social support received

Social support	N (%) of participants by weekly frequency of physical activity				
	Walking activity				p-value^*^
	0	1-3	4-5	6-7	
SS walking					0.719
Does not have support	12 (35.3)	9 (26.5)	10 (29.4)	3 (8.8)	
Has support	38 (31.9)	37 (31.0)	27 (22.7)	17 (14.4)	
SS moderate/vigorous					0.004
Does not have support	27 (49.1)	9 (16.4)	11 (20.0)	8 (14.5)	
Has support	24 (22.7)	38 (35.8)	28 (26.4)	16 (15.1)	
SS walking	**Moderate physical activity**				0.107
Does not have support	7 (20.6)	10 (29.4)	12 (35.3)	5 (14.7)	
Has support	17 (14.0)	49 (40.5)	23 (19.0)	32 (26.5)	
SS moderate/vigorous					0.207
Does not have support	14 (24.5)	18 (31.6)	14 (24.5)	11 (19.4)	
Has support	13 (12.3)	43 (40.6)	24 (22.6)	26 (24.5)	
SS walking	**Vigorous physical activity**				0.516
Does not have support	9 (26.5)	6 (17.6)	10 (29.4)	9 (26.5)	
Has support	20 (16.6)	18 (15.0)	47 (39.2)	35 (29.2)	
SS moderate/vigorous					0.156
Does not have support	17 (29.9)	9 (15.8)	16 (28.0)	15 (26.3)	
Has support	17 (16.3)	13 (12.5)	42 (40.4)	32 (30.8)	

* Fisher’s test.

SS walking = social support for walking; SS moderate/vigorous = social support for physical activity of moderate or vigorous intensity.

In Table 3, the results show associations between social support and frequency of physical activity. Workers who reported receiving social support for walking had a 1.22 times greater likelihood (OR: 1.22; 95%CI: 1.00-1.49) of a higher frequency of walking activity than people without social support for this activity. In contrast, social support for physical activity of moderate or vigorous intensity was not a predictor for increased frequency of moderate intensity physical activity (OR: 1.03; 95%CI: 0.83-1.26).

**Table 3:** Odds ratios (OR) and their respective 95% confidence intervals (95%CI) for associations between social support and weekly physical activity frequency

Social support	Weekly physical activity frequency					
	Walking OR (95%CI)		Moderate OR (95%CI)		Vigorous OR (95%CI)	
	Crude model	Adjusted model^*^	Crude model	Adjusted model	Crude model	Adjusted model
SS walking						
Does not have support	1.00	1.00	1.00	1.00	1.00	1.00
Has support	1.17 (0.96-1.43)	1.22 (1.00-1.49)^†^	0.99 (0.81-1.21)	1.03 (0.83-1.26)	1.06 (0.83-1.34)	1.07 (0.84-1.37)
SS moderate/vigorous						
Does not have support	1.00	1.00	1.00	1.00	1.00	1.00
Has support	1.25 (1.06-1.47)^†^	1.32 (1.11-1.58)^†^	1.09 (0.92-1.31)	1.12 (0.95-1.35)	1.29 (1.05-1.59)^†^	1.34 (1.08-1.67)^†^

* Adjusted model: sex, age, educational level.

† Statistically significant associations.

SS walking = social support for walking; SS moderate/vigorous = social support for moderate or vigorous intensity physical activity.

Still with relation to social support for moderate or vigorous activities, the workers who reported receiving social support exhibited a 1.32 times greater likelihood (OR: 1.32; 95%CI: 1.11-1.58) of an increased frequency of walking and 1.34 times greater likelihood (OR: 1.34; 95%CI: 1.08-1.67) of an increased frequency of vigorous activities.

## DISCUSSION

The objective of this study was to investigate the association between social support for physical activities and weekly frequency of physical activity in adult workers at a public university in the Brazilian state of Rio de Janeiro. Significant associations were detected between social support for both types of physical activity investigated (social support for walking and social support for moderate or vigorous physical activity) and weekly frequency of physical activity in different domains (walking and vigorous activity). Of the pathways linking social support to physical activity, Ayotte et al.^19^ suggest that the primary pathway acts indirectly via sociocognitive constructs, such as self-efficacy and motivation. Motivation has an important role to play, since it can reinforce habits, whether because of the health benefits or esthetics or even because of the pleasure that physical activity can provide. Self-efficacy acts through perception of personal and environmental barriers directly linked to behaviors, because being in contact with other people can influence the sense of one’s own ability to perform certain activities, irrespective of perceived barriers because of information and feedback provided, boosting readiness to perform physical activity.^20,21^

With relation to our findings, workers who reported receiving social support for walking were more likely to have greater weekly frequency. These results may reflect the setting in which these people work, since the university campus where these contract workers work is a large space that makes walking a viable means of transport. Additionally, since the instrument used to measure physical activity cannot distinguish the dimension of this behavior, it is possible that a significant proportion of walking physical activity reported by these participants is associated with their displacement.

With regard to moderate physical activity, the results did not reveal any association between social support for physical activities of moderate or vigorous intensity and frequency of moderate physical activity. In contrast, the psychosocial resource does appear to have an important influence on this behavior, since there are reports in the literature that people who feel encouraged by their social environment are more likely to meet physical activity recommendations than those who do not have a motivating environment.^22^ This highlights the role of social support in physical activity among adults and that the simple fact of being in contact with people who exercise is associated with an increase in physical activity,^23^ underscoring the relevance of encouragement from relatives, friends, and neighbors as a protection factor against low physical activity levels.

Participants who reported social support for moderate or vigorous activities exhibited higher frequency of vigorous physical activity, suggesting that they are surrounded by a network of relationships that tends to support physical activity of vigorous intensity. It is understandable that maintaining the frequency of physical activity may not be an easy task, especially among workers who spend a large part of their daily routines performing work activities and may have little time to dedicate to activities that exceed the resting state. Therefore, the ways in which this psychosocial resource is provided may provoke positive changes in the frequency of these behaviors.^24^ In analogy, authors who examined the characteristics of support networks associated with physical activities of moderate and vigorous intensity have observed that there is a greater likelihood of participation in physical activities when people have someone to help with routine chores or have company when performing the activity.^25^

Social support for moderate and vigorous physical activity was also associated with the frequency of walking activity. These findings are in agreement with those of Oliveira et al.,^21^ who investigated how four dimensions of social support affect engagement, maintenance, activity type, and time spent in leisure-time physical activity among public sector, non-faculty workers at a university in Rio de Janeiro, during a 2-year follow-up period. They observed that people with high levels of support in the positive social interaction dimension were more likely to perform more than 4 hours per week of physical activity, compared with other people who performed 2 hours or less per week, showing that the positive social interaction dimension significantly increased the likelihood of a person coming into contact with others and becoming involved in physical activities. This culminates in expanding the network of relationships, increasing the supply of social support for physical activity.

Previous studies have identified associations between certain dimensions of social support and the time dedicated to physical activity,^26,27^ since, as social support is a protective resource resulting from a perceived threat and the desire to avoid certain potential negative behavioral responses and as the positive social interaction dimension (such as having someone with whom to do pleasurable activities, for example) involves social control via norms and attitudes, this construct may therefore be related to more time dedicated to this activity in circumstances in which the social network provides social support.^21^ With regard to the frequency of physical activity, little is known about the true relationship, since studies generally investigate social support in relation to people’s physical activity levels.^28,29^

Certain limitations of this study should be mentioned. Data were only collected once, so the temporal relationships between events cannot be explained. There is therefore no way to identify causal relationships. Although the instrument employed to investigate social support for physical activity offers the possibility of differentiating social support for walking from social support for other physical activities, it cannot distinguish between social support for moderate and vigorous intensity physical activities, curtailing more detailed analyses and interpretations. Other relevant features are the sample size and the use of a sample of convenience, since the representativeness of the study population cannot be guaranteed, limiting extrapolation of the results to other working populations.

Although the instrument used in its short form to measure physical activity is widely employed, allowing for comparisons between different studies, it is subjective, which increases the margin of error when compared to objective measures, since studies reveal that people tend to overestimate their physical activity, which is a socially desirable behavior.^30^

## CONCLUSIONS

In view of the relevance of physical activity to health and since the choice to adopt healthy habits is linked to economic, emotional, and, especially, social factors, it is crucial to achieve better understanding of the influence of the social environment on this behavior. The results of the present study investigating the association between social support and weekly physical activity frequency support the conclusion that performing physical activity together with friends and family, their encouragement, and their invitations to participate are all associated with the frequency of physical activity. However, the impact of social support for physical activity of moderate and vigorous intensity appears to be more effective for increasing the frequency of vigorous activities than for other intensities. Longitudinal studies are needed to investigate the effect of social support on weekly physical activity frequency in different scenarios.
